# Catestatin, vasostatin, cortisol, and pain assessments in dogs suffering from traumatic bone fractures

**DOI:** 10.1186/s13104-017-2450-y

**Published:** 2017-03-21

**Authors:** Thanikul Srithunyarat, Ragnvi Hagman, Odd V. Höglund, Mats Stridsberg, Ulf Olsson, Jeanette Hanson, Chalermkwan Nonthakotr, Anne-Sofie Lagerstedt, Ann Pettersson

**Affiliations:** 1 Department of Clinical Sciences, Swedish University of Agricultural Sciences, Box 7054, 75007 Uppsala, Sweden; 2 Faculty of Veterinary Medicine, Khon Kaen University, Khon Kaen, 40002 Thailand; 3 Department of Medical Sciences, Uppsala University, 75185 Uppsala, Sweden; 4 Unit of Applied Statistics and Mathematics, Swedish University of Agricultural Sciences, Box 7013, 75007 Uppsala, Sweden

**Keywords:** Biomarker, Chromogranin A, Morphine analgesia, Stress response

## Abstract

**Background:**

Traumatic bone fractures cause moderate to severe pain, which needs to be minimized for optimal recovery and animal welfare, illustrating the need for reliable objective pain biomarkers for use in a clinical setting. The objectives of this study were to investigate catestatin (CST) and vasostatin (VS) concentrations as two new potential biomarkers, and cortisol concentrations, scores of the short form of the Glasgow composite measure pain scale (CMPS-SF), and visual analog scale (VAS) in dogs suffering from traumatic bone fractures before and after morphine administration in comparison with healthy dogs.

**Methods:**

Fourteen dogs with hind limb or pelvic fractures and thirty healthy dogs were included. Dogs with fractures were divided into four groups according to analgesia received before participation. Physical examination, CMPS-SF, pain and stress behavior VAS scores were recorded in all dogs. Saliva and blood were collected once in healthy dogs and in dogs with fractures before and 35–70 min after morphine administration. Blood samples were analyzed for CST, VS, and cortisol. Saliva volumes, however, were insufficient for analysis.

**Results:**

Catestatin and cortisol concentrations, and CMPS-SF, and VAS scores differed significantly between dogs with fractures prior to morphine administration and healthy dogs. After morphine administration, dogs with fractures had significantly decreased CMPS-SF and VAS scores and, compared to healthy dogs, CST concentrations, CMPS-SF, and VAS scores still differed significantly. However, CST concentrations remained largely within the normal range. Absolute delta values for CST significantly correlated with delta values for CMPS-SF. Catestatin and cortisol did not differ significantly before and after morphine administration. Vasostatin concentrations did not differ significantly between groups.

**Conclusions:**

Catestatin and cortisol concentrations, CMPS-SF, and VAS scores differed significantly in the dogs with traumatic bone fractures compared to the healthy dogs. Morphine treatment partially relieved pain and stress according to the subjective but not according to the objective assessments performed. However, because of the large degree of overlap with normal values, our results suggest that plasma CST concentrations have a limited potential as a clinically useful biomarker for pain-induced stress.

## Background

Traumatic bone fractures occur commonly in dogs, often requiring both analgesia and surgery as a part of the treatment. The pain level induced by bone fractures is reported to be moderate to severe and opioids have been shown to be the analgesia of choice [1, 2]. It is essential to provide sufficient pain relief for animal welfare reasons, and to reduce recovery time and duration of hospitalization [3]. The perception of pain varies between individuals [4]. Therefore, proper assessment of each patient is necessary for optimal analgesia administration. However, pain evaluation is a challenging task because all currently available assessment methods have limitations, and no one method has been found to be optimal for all animals [2, 5].

Pain evaluation can be performed by using subjective assessment methods, e.g. observation of behaviors, and by measuring objective physiological and hormonal parameters that are influenced by a neuroendocrine stress response. Several subjective pain assessment tools have been used in dogs and cats: for example, simple descriptive scores (SDS), numeric rating scales (NRS), visual analog scales (VAS), Glasgow composite measure pain scales (CMPS), and short form of the Glasgow composite measure pain scales (CMPS-SF) [5–10]. The CMPS and the CMPS-SF combine several subjective behaviors for assessment of acute pain, and both have been validated for use in dogs [8, 11].

Several physiological parameters change in response to activation of a neuroendocrine stress response characterized by stimulation of the hypothalamic-pituitary-adrenal (HPA) axis and the sympatho-adrenal-medullary (SAM) axis [12, 13] such as heart rate, respiratory rate, blood pressure, and blood glucose, cortisol, and catecholamines concentrations [14]. Although unspecific, these parameters have long been used in studies on pain and stress response in animals, alone or in combination with other parameters [13, 15, 16].

Measurement of cortisol has traditionally been used as an indicator for increased stress or pain. However, in addition to stress, plasma cortisol concentrations vary considerably over time due to pulsatile secretion, and there may be an influence from circadian rhythm [17–20]. Therefore, the usefulness of plasma cortisol as a sole biomarker in a clinical setting is limited. Still, until better biomarkers are found, measurement of cortisol concentrations remains important in studies on stress [21, 22].

Although catecholamines increase promptly after SAM stimulation, they have a short half-life and are rapidly degraded, which limits their usefulness in a clinical setting [23]. Chromogranin A (CgA) has recently been suggested as an alternative marker for SAM activation. Chromogranin A is a glycoprotein, which is co-released with catecholamines and other neuroendocrine hormones such as adrenaline, noradrenaline, dopamine, and neuropeptides [24, 25]. Concentration of CgA is rather stable for handling and storage compared to catecholamines with longer circulating half-life of 18 min [25–27].

CgA concentrations are increased in patients with enterochromaffin-like cell hyperplasia in the gastric mucosa which can be seen in atrophic gastritis with *Helicobacter pylori* infection, and in conjunction with proton pump inhibitor treatment [28–30]. In the absence of neuroendocrine tumors and gastrointestinal disease, plasma CgA concentration is considered to reflect the activity of the SAM axis. In human studies, CgA measurements in saliva and plasma have shown promise as potential biomarkers for stress [31–39]. In dogs, only a few studies on CgA have so far been published [40–47]. Chromogranin A can be measured in dogs and cats by estimating the CgA epitopes catestatin (CgA 361–372; CST) and vasostatin (CgA 17–38; VS) using radioimmunoassay (RIA) [43]. We have previously established normal reference ranges for plasma CST and VS using RIA in healthy dogs accustomed to sampling procedures and investigated the correlation between CST and VS [48]. Plasma CST and VS and saliva CST concentrations have been shown not to be affected by circadian, age, gender, or breed variations [41, 48].

In order to evaluate the usefulness of the CgA epitopes CST and VS as clinical biomarkers for pain-induced stress in dogs, studies performed in dogs that are experiencing acute pain are needed. The objectives of this study were to investigate CST and VS concentrations as two new potential biomarkers, and cortisol concentrations, scores of the CMPS-SF, and VAS in dogs suffering from traumatic bone fractures before and after morphine administration in comparison with healthy dogs. We hypothesized that the concentrations of CST and VS should differ between healthy dogs and dogs with traumatic bone fractures before as well as after morphine administration. Furthermore, we hypothesized that the changes in CST and VS concentration would agree with other stress and pain assessments.

## Methods

### Study design and ethical approval

This study was designed as a prospective clinical study and was approved by Khon Kaen University Ethical Legislation (AEKKU 26/2557). The study was performed during March to June 2015. The dog owners were informed and gave their consent prior to participation. All dogs were admitted and treated according to the routines at Khon Kaen University (KKU) Veterinary Teaching Hospital, Khon Kaen, Thailand. Participation in this study did not, in any manner, delay morphine administration to the dogs with bone fractures.

### Dogs

#### Dogs with traumatic bone fractures

All dogs in the study were client-owned. Both primary and referral cases with traumatic bone fractures limited to the hind limbs and pelvis were included. On the day of admission, a standardized complete physical examination was performed in all dogs. Blood screening (hematology and blood biochemistry) for evaluation of the overall health status was performed according to the routines at KKU Veterinary Teaching Hospital [46]. Additionally, the dogs’ health status were classified in accordance with the American Society Anesthesiologists (ASA) Physical Status Classification System [49, 50]. Dogs with traumatic ASA scores exceeding II, other concurrent disease, or that had received corticosteroid or proton pump inhibitor therapy were excluded from the study.

All dogs with bone fractures that fulfilled the inclusion criteria and, based on history taking at the time of admission, had a time lapse up to 4 days from injury and a withdrawal period from previous analgesia over 6 h were included. Dogs were grouped, according to cause of trauma, duration of fracture, fracture site and number of bone fractures, previous analgesia, and withdrawal period from previous analgesia. Dogs with traumatic bone fractures received a prompt paralumbar intramuscular injection with 0.5 mg/kg morphine sulfate (Morphine Sulfate injection, M & H manufacturing, Samutprakan, Thailand). Blood and saliva samples were collected immediately before and 35–70 min after morphine administration.

#### Healthy dogs

Client-owned healthy dogs admitted for elective ovariohysterectomy were included as a control group. Data from these dogs were obtained from a previous study on CST and VS in dogs undergoing ovariohysterectomy [46]. Preoperative physical examination and blood screening were performed, in the same manner as for the dogs with bone fractures, to confirm a healthy status prior to inclusion in the study. Only dogs with ASA scores I with no other concurrent disease and that had not received steroid or proton pump inhibitor therapy were included. Samples and data were collected prior to surgery and preoperative medication on the day of surgery as previously described [46].

### Method and sample protocol

The physical examination, assessments of CMPS-SF and overall pain score using VAS (OP-VAS), saliva and blood sample collection, and scoring of stress behavior using VAS (S-VAS) were performed on each sampling occasion. The order of sample collection was randomized, with an interval between saliva and blood sampling of less than 5 min. In dogs with fractures, blood and saliva samples were collected immediately before and 35–70 min after morphine administration, as earlier described. All sampling and assessments included in the study were performed prior to any surgery. In healthy dogs, samples were collected once before any premedication on the day of surgery.

### Subjective pain and stress assessment

In association with each sample collection, pain was scored using the CMPS-SF [11] and VAS for pain (OP-VAS), in which one end of the scale indicated no pain and the other end referred to the worst possible pain. In dogs with traumatic bone fractures, section B of the CMPS-SF was not performed because all dogs had mobility problems and, therefore, the total score of the CMPS-SF was 20. A score of 5 or more indicated the need of analgesia.

**Fig. 1:**
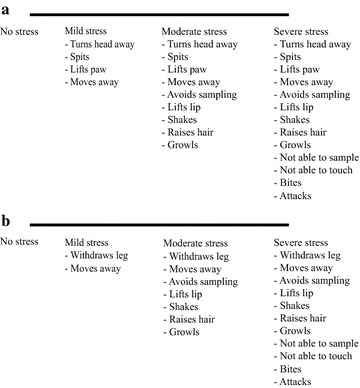
Stress behavior visual analog scale (S-VAS). 1**a** Criteria used for evaluation of stress behavior during saliva sampling. 1**b** Criteria used for evaluation of stress behavior during blood sampling. The criteria of the S-VAS [48] was modified from Norling et al. [51]

The stress behavior level was assessed during each saliva and blood sampling occasion using S-VAS [48] by observing avoidance behaviors during sample collection. Pre-established criteria, modified from Norling et al. [51], were used for determination of stress levels during sampling (as illustrated in Fig. 1). All subjective pain and stress assessments were performed by the same observer (TS).

### Saliva and blood collection

Saliva was collected using a swab, sized 8 × 125 mm, (SalivaBio Children’s swab, Salimetrics, PA, USA) which was placed into the buccal cavity for 90 s. The swab was thereafter transferred to a 17 × 100 mm swab storage tube (Swab storage tubes, Salimetrics, PA, USA) and centrifuged at 3000 rpm (1401*g*) for 15 min. The swab was subsequently removed and the saliva deposit stored at −20 °C.

Blood samples were collected from the distal cephalic vein using butterfly needles (BD Vacutainer, Becton-Dickson, Plymouth, United Kingdom) into vacuum lithium heparin tubes and clot activator tubes (BD Vacutainer, Becton-Dickson, Plymouth, United Kingdom). The tubes were centrifuged at 3300 rpm (1695 g) for 5 min. The heparinized plasma and serum obtained was transferred into cryotubes (Low Temperature Freezer Vials, VWR, Stockholm, Sweden) and stored at −20 °C.

After the clinical part of the study was completed, all samples were transported to the Swedish University of Agricultural Sciences (SLU), Uppsala, Sweden, by a private transportation company (Temperature control, World Courier, Bangkok, Thailand), and arrived within 48 h. During transportation, the temperature was controlled, monitored and remained below −20 °C. After arrival at SLU, the samples were freeze stored at −70 °C until analysis within a maximum of 7 months after collection.

### Catestatin and vasostatin analysis

Concentrations of CST and VS were analyzed in saliva and plasma samples using rabbit antibodies against the human CgA amino acid sequence 17–38 for VS and sequence 361–372 for CST. Samples were analyzed in duplicate by competitive RIA at the Clinical Chemistry Laboratory, Uppsala University Hospital, Uppsala, Sweden as earlier described [43]. The overall coefficient of variation (CV) in this study was <10%.

### Cortisol analysis

Serum cortisol concentrations were analyzed in duplicate. The analysis, using a solid-phase competitive chemiluminescent enzyme immunoassay (Immulite 2000, Siemens, Erlangen, Germany), was performed at the Clinical Chemistry Laboratory, University Animal Hospital, SLU, Uppsala, Sweden. The intraassay CV was <5%. The limit of detection for cortisol analysis was 10 nmol/L. Samples with a concentration lower than the detection limit were recorded as 5 nmol/L.

### Statistical analysis

In all analyses, residuals were checked for normality and homoscedasticity using diagnostic plots. Because the plasma VS values appeared skewed, this variable was log transformed (natural log) prior to analysis. For all variables, comparisons between healthy dogs and dogs with traumatic bone fractures were made using independent samples t tests. Comparisons of values before and after morphine administration in dogs with traumatic bone fractures were made using mixed linear models with “dog” as a random factor; this is analogous to a paired *t* test. Pairwise comparisons were adjusted for multiplicity using Tukey’s method. Similar analyses were also made to assess the effects of analgesia received before participation in the study, cause of trauma (unknown or car accident), duration of fracture (<48 or ≥48 h), site (femur, tibia, both femur and tibia, or pelvis) and number of bone fractures (1–7), and withdrawal period from previous analgesia (≤12 or >12 h).

Delta values for CMPS-SF, OP-VAS, serum cortisol, and plasma CST concentrations were calculated by subtracting post treatment values/scores from baseline values prior to morphine treatment. Delta values without + or − signs were defined as absolute delta values. The correlation of analgesia group, the delta values for CMPS-SF, OP-VAS scores and the absolute delta values for serum cortisol and plasma CST concentrations were calculated using Proc corr.

All analyses were performed using SAS (2015) software [52]. Respiratory rates for panting dogs were recorded as 200 per minute. The selected level of significance was *p* < 0.05.

## Results

Fourteen intact dogs with bone fractures limited to the hind limbs and pelvis and thirty intact healthy dogs admitted for elective ovariohysterectomy were included in the study. Mean ± SD age, body weight, and body condition scores of the included dogs are illustrated in Table 1.

**Table 1:** Age, weight, and body condition scores in 14 dogs with fractures and 30 healthy dogs

Parameters	Dogs with traumatic bone fractures (n = 14)	Healthy dogs (n = 30)
Age (months)	12 ± 16	28 ± 26
Body weight (kg)	19.0 ± 9.7	11.6 ± 7.0
Body condition score (9 grade scale)	6 ± 1	5 ± 1

Data presented as mean ± SD

The healthy dogs were all females of different breeds including Chihuahua (n = 2), Thai Ridgeback (n = 1), Thai Bangkaew (n = 1), Pomeranian (n = 3), Shih Tzu (n = 1), Maltese (n = 1), Siberian Husky (n = 1), Labrador Retriever (n = 1), Poodle (n = 1), and Mixed Breeds (n = 18). Five of the dogs with fractures were females and nine males of different breeds including Pitbull (n = 2), Golden Retriever (n = 3), Labrador Retriever (n = 1), Pomeranian (n = 1), and Mixed Breeds (n = 7). The fractures were caused by traffic accidents (n = 12) or by unknown trauma (n = 2). The average (mean ± SD) duration of bone fracture injury prior to inclusion in the study was 46 ± 29 h. The fracture location was femur in seven dogs (one fracture in five dogs and two fractures in two dogs), tibia in four dogs (one fracture in three dogs and two fractures in one dog), both femur and tibia in one dog (in total two fractures), and pelvis in two dogs (five and seven fractures respectively). They were allocated into four groups according to analgesia received before participation as follows; no analgesia (n = 3), unknown/not specified analgesia (n = 3), carprofen (n = 4), or morphine (n = 4) (Table 2). The general attitude from the physical examinations varied from responsive (n = 40) to depressed (n = 4). The blood screening results in the dogs with bone fractures showed leukocytosis with mild neutrophilia (n = 7) whereas the results in the healthy dogs were all within the reference ranges.

**Table 2:** Previous analgesia received, duration of bone fractures, withdrawal period after previous analgesia, and cortisol concentration

Analgesia group	Duration of bone fractures (hours)	Analgesia withdrawal period (hours)	Cortisol concentration (nmol/L)
None^a^ (n = 3)	24, 24 (1, 24, 48)		18.1 ± 22.6
Unknown^b^ (n = 3)	48, 48 (25, 48, 72)	22, 24 (15, 24, 28)	99.4 ± 48.0
Carprofen^c^ (n = 4)	62, 60 (30, 48, 72, 96)	21, 21 (12, 18, 24, 28)	157.3 ± 41.5
Morphine^d^ (n = 4)	47, 36 (18, 24, 48, 96)	14, 16 (6, 13, 19, 19)	133.0 ± 69.3

Prior to inclusion, dogs with bone fractures were allocated into four groups according to analgesia received before participation (minimum 6 h withdrawal period prior to first sampling) as indicated above. Data presented as mean ± SD of serum cortisol concentration and mean, median (individual data) of duration of bone fractures and analgesia withdrawal period

^a^No analgesia received

^b^No available information of type of drug and dose

^c^Dogs received carprofen 2.2 mg/kg twice daily to 4.4 mg/kg once daily subcutaneous or orally

^d^Dogs received morphine 0.5 mg/kg intramuscular or subcutaneous

Mean ± SD of the CMPS-SF, OP-VAS, and saliva and blood sampling S-VAS scores, temperature, heart rate, respiratory rate, concentrations of serum cortisol, and plasma CST and VS in dogs with traumatic bone fractures before and after morphine administration and in the healthy dogs are shown in Table 3.

**Table 3:** Pain assessment results in 14 dogs with bone fractures and 30 healthy dogs

Parameters	Healthy dogs (n = 30)	Traumatic bone fracture dogs (n = 14)	
		Before morphine	After morphine
The CMPS-SF (/20)^a^	0	5.9 ± 2.6	3.9 ± 2.1
OP-VAS (mm)^a^	0	40.1 ± 13.7	33.3 ± 12.8
Saliva sampling S-VAS (mm)^a^	41.6 ± 23.5	28.1 ± 10.6	20.3 ± 7.6
Blood sampling S-VAS (mm)^a^	37.7 ± 22.1	22.0 ± 11.6	14.2 ± 9.3
Temperature (°C)^b^	38.9 ± 0.4	38.7 ± 0.5	38.1 ± 0.5
Heart rate (beats/minute)^c^	123.9 ± 30.6	137.1 ± 32.9	108.6 ± 24.3
Respiratory rate (breaths/minute)	92.6 ± 63.3	100.9 ± 59.6	117.9 ± 63.3
Serum cortisol (nmol/L)^d^	174.6 ± 78.5	108.1 ± 69.0	130.9 ± 93.6
Plasma catestatin (nmol/L)^e^	0.76 ± 0.17	0.61 ± 0.15	0.58 ± 0.16
Plasma vasostatin (nmol/L)	1.12 ± 2.16	0.39 ± 0.12	0.39 ± 0.07

*CMPS-SF* short form of the Glasgow composite measure pain scale, *OP-VAS* score of visual analog scale for overall pain, *S-VAS* stress behavior visual analog scale during saliva and blood sampling. Significantly different using Tukey adjustment when *p* < 0.05

^a^Levels significantly differed in dogs with bone fractures and healthy dogs (between healthy dogs and dogs with fractures before morphine treatment, *p* < 0.0001, *p* < 0.0001, *p* = 0.048, and *p* = 0.02 for CMPS-SF, OP-VAS, saliva and blood sampling S-VAS, respectively, and between healthy dogs and dogs with fractures after morphine treatment, *p* < 0.0001, *p* < 0.0001, *p* = 0.002, and *p* = 0.0005 for CMPS-SF, OP-VAS, saliva and blood sampling S-VAS, respectively), and also before and after morphine administration (*p* = 0.045, *p* = 0.02, *p* = 0.02, and *p* = 0.01 for CMPS-SF, OP-VAS, saliva and blood sampling S-VAS, respectively)

^b^Temperature in dogs with bone fractures after morphine administration differed significantly from either before morphine administration (*p* = 0.0008) or healthy dogs (*p* < 0.0001). No difference was found between dogs with fracture before morphine administration and healthy dogs

^c^Heart rate in dogs with bone fractures after morphine administration differed significantly from before morphine administration (*p* = 0.001). No difference was found between healthy dogs and dogs with fracture before and after morphine administration

^d^Serum cortisol levels in dogs with fractures before morphine injection differed significantly from healthy dogs (*p* = 0.01). No difference was found between dogs with fractures before and after morphine treatment and between after morphine treatment and healthy dogs

^e^Plasma catestatin levels in dogs with bone fractures before (*p* = 0.009) and after morphine administration (*p* = 0.002) differed significantly from levels in healthy dogs. No difference was found between before and after morphine administration in the dogs with fractures

The CMPS-SF scores in healthy dogs were zero whereas in dogs with bone fractures before receiving morphine they were over 5/20 indicating pain. Prior to morphine administration, in dogs with traumatic bone fractures, CMPS-SF and OP-VAS scores were significantly higher than in healthy dogs (*p* < 0.0001). After morphine administration, in dogs with fractures, the CMPS-SF and OP-VAS scores were significantly decreased compared to before morphine administration (*p* = 0.005 and 0.02, respectively), however, scores were significantly higher compared to healthy dogs (*p* < 0.0001).

Saliva and blood S-VAS scores in dogs with fractures were significantly lower than the healthy dogs, both before (*p* = 0.048 for saliva S-VAS and *p* = 0.02 for blood S-VAS) and after morphine administration (*p* = 0.002 for saliva S-VAS and *p* = 0.0005 for blood S-VAS). Saliva and blood S-VAS scores were significantly decreased in dogs with fractures after morphine administration compared to before (*p* = 0.02 for saliva S-VAS and *p* = 0.01 for blood S-VAS).

In dogs with fractures, body temperature and heart rate significantly decreased after morphine administration compared to before (*p* = 0.0008 for temperature and *p* < 0.0001 for heart rate) and in healthy dogs (*p* < 0.0001 for both temperature and heart rate). Temperature and heart rate did not significantly differ between healthy dogs and dogs with fractures prior to morphine administration. Respiratory rate did not differ significantly at any time point.

Prior to morphine administration, in dogs with traumatic bone fractures, serum cortisol concentrations were significantly lower than healthy dogs (*p* = 0.01). Serum cortisol concentrations in dogs with fractures, before morphine administration, and in healthy dogs did not differ significantly after morphine administration.

Plasma CST concentrations, in dogs with fractures both before (*p* = 0.009) and after (*p* = 0.002) morphine administration, were significantly lower compared to healthy dogs. No significances in plasma CST concentrations were found between before and after morphine administration. Plasma VS did not differ significantly at any time point. The volume of saliva was insufficient for analysis of both CgA epitopes in the dogs with traumatic bone fractures, and saliva CST could only be analyzed in 12 of the samples from healthy dogs. Therefore, saliva CST and VS could not be compared between these two dog groups.

**Fig. 2:**
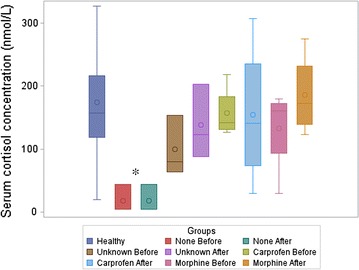
Serum cortisol in 30 healthy and 14 dogs with fractures before and after morphine administration. *Dogs without previous analgesia (None, n = 3) prior to inclusion to the study had significantly (*p* = 0.02) lower cortisol concentration than dogs in the other analgesia groups. No significant difference was found between unknown (Unknown, n = 3), carprofen (Carprofen, n = 4), or morphine (Morphine, n = 4) analgesia groups. No significant difference in cortisol concentration was found between before (Before) and after (After) morphine administration in dogs with traumatic bone fractures (n = 14). The *upper vertical line* (*whisker*) refers to maximal value and *lower whisker* refers to minimal value. The length of the box refers to interquartile range from quartile 1–3 (percentile 25–75) where the *top* of the box is quartile 3 and the *bottom* is quartile 1. The *circle* symbol in the box refers to mean and the *horizontal line* in the box is median

**Fig. 3:**
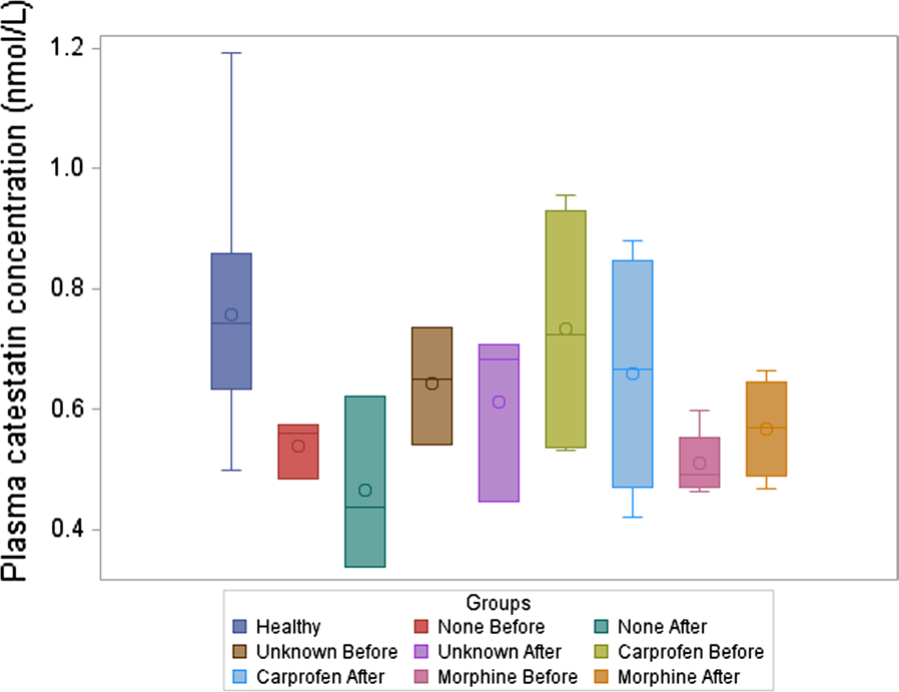
Plasma catestatin in 30 healthy and 14 dogs with fractures before and after morphine administration. No significant difference was found between the analgesia groups (None, Unknown, Carprofen, Morphine) or when compared before (Before) and after (After) morphine administration in the dogs with traumatic bone fractures. The *upper vertical line* (*whisker*) refers to maximal value and *lower whisker* refers to minimal value. The length of the box refers to interquartile range from quartile 1–3 (percentile 25–75) where the *top* of the box is quartile 3 and the *bottom* is quartile 1. The *circle* symbol in the box refers to mean and the *horizontal line* in the box is median

**Fig. 4:**
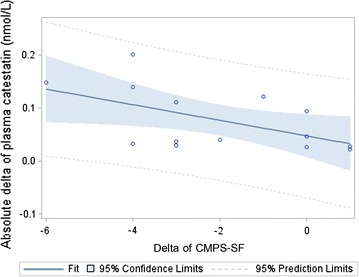
Absolute delta values for plasma catestatin concentrations in relation to delta values for CMPS-SF. In dogs with bone fractures (n = 14), the absolute delta values for plasma catestatin correlated significantly to the delta values for CMPS-SF (*p* = 0.04, r = 0.31). Dogs with lower delta values for CMPS-SF scores, indicating good pain relief, had higher absolute delta values for plasma catestatin. *CMPS-SF* the short form of Glasgow composite measure scale

As previously stated, the dogs were allocated into four groups according to analgesia received before participation as follows; no analgesia (n = 3), unknown/not specified analgesia (n = 3), carprofen (n = 4), or morphine (n = 4) (Table 2). Dogs that had not received any analgesia before inclusion had initially significantly lower serum cortisol concentrations (*p* = 0.02) compared to dogs in the other analgesia groups, whereas no significant difference was found between the unknown, carprofen, and morphine analgesia groups (Fig. 2). Plasma CST concentrations did not differ (*p* = 0.31) between the different analgesia groups (Fig. 3). There were no significantly changes in concentrations of serum cortisol and plasma CST before and after morphine administration regardless of analgesia group (Figs. 2, 3). The absolute delta values for serum cortisol and plasma CST did not significantly correlated with analgesia group or with the delta values of OP-VAS. The absolute delta values for plasma CST, however, significantly correlated with the delta values for CMPS-SF (*p* = 0.04, r = 0.31) (Fig. 4). Cause of injury, duration of fracture, site and number of bone fractures, and withdrawal period from previous analgesia had no effect on any of the investigated variables in the study.

## Discussion

In this study, dogs with traumatic fractures of the hind limbs or pelvis exhibited increased pain, compared to the control dogs, as measured by the CMPS-SF and OP-VAS. The pain was reduced after morphine administration. Although both circulating cortisol and CST levels differed significantly between the injured and healthy dogs in this study, no significant change was detected after morphine administration and plasma CST levels overlapped with the previously established normal range [48].

The significantly higher subjective pain scores seen in dogs with traumatic bone fractures compared to the control dogs was an expected finding because pain and stress can induce behavioral changes [7, 8, 53]. The dogs with fractures had an average CMPS-SF score of ≥5/20 and OP-VAS over 40 mm, which, according to what has previously been reported, indicates pain and the need for additional analgesic treatment [11, 54]. Surprisingly, the S-VAS scores measured during collection of saliva and blood samples were significantly lower in dogs with traumatic bone fractures compared to the healthy dogs. The S-VAS scores used, however, were based on avoidance behavior during sample collection and thus the decreased avoidance behavior seen in the dogs with fractures in this study may reflect the dog’s general condition more than experienced stress and pain.

The subjective pain assessments decreased significantly after morphine administration indicating decreased pain perception. The level of pain may have been underestimated because of the sedative effect of morphine affecting some of the parameters [54, 55]. However, despite receiving morphine analgesia, the subjective pain assessment scores were still higher in dogs with fractures than in the healthy dogs, indicating a suboptimal analgesic effect. Traumatic bone fractures may require multimodal analgesia and single morphine injections are often insufficient [2, 5, 56, 57]. The administration route and circulatory competency of the patient may affect the bioavailability of morphine sulfate. Peak plasma concentrations of morphine sulfate, after intramuscular injection in dogs, has been shown to occur within 5 min with a half-life of 82 min [56], and our samples were collected within this interval. Because none of the dogs in our study showed any clinical signs of circulatory incompetence, the finding of inadequate pain relief in this study was probably an effect of dosage, i.e. a too low dose administered. These findings illustrate the importance of repeated pain assessments during the convalescent period to be able to adjust the analgesic therapy based on what is needed.

Routinely measured physiological parameters, such as body temperature, heart rate, and respiratory rate were similar in dogs with bone fractures and in the healthy dogs in this study. This was an unexpected finding; however, these physiological parameters are unspecific and can be affected by many factors such as environmental temperature and various surrounding stressful conditions like a visit to a veterinary clinic [15, 16]. Body temperature and heart rate decreased significantly after morphine administration. This could indicate reduced pain levels, but morphine may have side-effects such as cardiovascular and respiratory depression, hypothermia, hypotension, sedation, anxiety, and bradycardia [58] which further illustrates the limitation of these physiological parameters for monitoring pain.

In the present study, cortisol concentrations were significantly lower in dogs with traumatic bone fractures prior to morphine administration compared to the healthy dogs. Furthermore, when comparing the different initial analgesic groups, cortisol concentrations in the dogs that had not received analgesia before inclusion were significantly lower than in the dogs that had received analgesia prior to inclusion. These results may reflect a downregulation of the HPA axis caused by long-standing pain in the dogs with fractures, leading to subsequent low concentrations of cortisol, which has been shown previously in studies of chronic pain and stress disorders [20, 59–61].

After morphine administration, the absolute delta values for and concentrations of cortisol did not change significantly. It was initially expected that cortisol concentration would normalize after morphine analgesia [59, 62], however, the CMPS-SF and OP-VAS, as stated previously, indicated suboptimal analgesia obtained in the dogs with fractures. In addition, there are other potential contributing factors that may influence the serum cortisol concentrations. For example, the episodic and pulsatile secretion of cortisol which limits the usefulness of a single measurement of plasma cortisol because it is unknown if the sampling is taken at a peak or trough in plasma cortisol concentrations [18–20, 63]. Psychologically induced stress from being kept at the animal clinic may also limit serum cortisol’s usefulness as a pain biomarker in a clinical setting [15, 16, 64, 65].

Plasma VS concentrations did not change significantly between any time points in this study, whereas plasma CST concentrations, similar to serum cortisol, were significantly lower in dogs with traumatic bone fractures compared to the healthy control group. Catestatin has an inhibitory role as a negative feedback for the release of catecholamines and CgA [66], which may contribute to the decreased concentrations of plasma CST seen in this study. Decreased concentrations of CST have also been reported in dogs after ovariohysterectomy [46]. Dogs with lower delta values for CMPS-SF scores, indicating good pain relief, had higher absolute delta values for plasma CST. Although the absolute delta values for plasma CST correlated significantly to the delta values for CMPS-SF, the correlation was very weak and plasma CST and VS concentrations overlapped to a large degree with previously established normal ranges in dogs. In addition, cause of injury, duration of fracture, site and number of bone fractures, and withdrawal period from previous analgesia did not significantly affect plasma CST and VS concentrations in this study. In conclusion, although requiring further controlled experimental studies, repeated sampling of plasma CST may be of interest for evaluating pain progression within the same patient. However, the large degree of overlap with normal values indicates that plasma CST has limited potential as a single clinical biomarker for pain induced stress in dogs. In addition, our results showed that plasma VS is not useful for this purpose.

This study was performed as a clinical trial and several limitations should be addressed. Ideally, all dogs should have had the same type of fractures and duration of injury, however, because studies on pain in a clinical situation are difficult to standardize, this was not possible. However, in agreement with the 3Rs (replace, reduce, refine) for ethical use of animals in research, we chose to perform this first study on CST and VS’s potentials in a clinical setting despite these difficulties. By first investigating the potential of CST and VS as biomarkers in injured patients admitted to an animal hospital for treatment, we may reduce the need for unnecessary painful experimental studies.

As no data on these biomarkers were available in dogs with acute pain prior to this study, we were not able to determine sample size beforehand which was a limitation. This study was therefore designed as a cross sectional study. The control dogs included in this study were all intact females which is a limitation because gender may affect neuroendocrine secretion i.e. cortisol. However, we have previously shown that CST and VS are not affected by gender [48]. Samples should ideally have been collected in dogs of similar age, gender, and at the same time of day, which was not possible in a clinical setting within a reasonable time span. It would have been preferable if sedation scores had been evaluated. Subjective pain and stress assessment should be performed by a trained single observer as in this study [67], however, a blinded observer would have been preferable in order to reduce confounding effects. The episodic secretion of cortisol might be a confounding factor, however, whether or not there is a circadian variation in dogs is still controversial [18, 19]. Concentrations of CST and VS have previously been reported to be unaffected by age, gender, breed, and time of collection [48]. Several factors such as variations in the duration and severity of injury, previous analgesia administered, the analgesia withdrawal period, individual variation, and psychological stress may also affect the findings reported here [15, 62]. However, values of CST and VS overlapped to a large degree with the ranges previously established in healthy dogs accustomed to the sample procedure regardless of fracture, previous analgesia, and duration of injuries, and by the CMPS-SF and OP-VAS indicating pain. Our results, therefore, show that the potential of these CgA epitopes as biomarkers for pain-induced stress in dogs is limited in a clinical setting.

## Conclusions

Measurement of circulating cortisol and CST, as well as the CMPS-SF, OP-VAS, S-VAS scores, were used for pain assessment in dogs with traumatic bone fractures. Subjective, but not objective assessments, indicated that intramuscular morphine administration resulted in partially reduced pain in dogs with bone fractures. Plasma CST and VS remained to a large degree within the established reference ranges throughout the study indicating their limited use as biomarkers for pain-induced stress in dogs in this clinical setting.

## Abbreviations

CgA: chromogranin A
CMPS: Glasgow composite measure pain scale
CMPS-SF: short form of the Glasgow composite measure pain scale
CST: catestatin
CV: coefficient of variation
OP-VAS: overall pain behavior visual analog scale
RIA: radioimmunoassay
RPM: revolutions per minute
S-VAS: stress behavior visual analog scale
VS: vasostatin
VAS: visual analog scale
